# The Gas6/TAM System and Multiple Sclerosis

**DOI:** 10.3390/ijms17111807

**Published:** 2016-10-28

**Authors:** Mattia Bellan, Mario Pirisi, Pier Paolo Sainaghi

**Affiliations:** 1 Department of Translational Medicine, Università del Piemonte Orientale, UPO, via Solaroli 17, 28100 Novara, Italy; bellanmattia@yahoo.it (M.B.); mario.pirisi@med.uniupo.it (M.P.); 2 Immuno-rheumatology Unit, Internal Medicine Division, “Maggiore della Carità” Hospital, 28100 Novara, Italy; 3 IRCAD, Interdisciplinary Research Center of Autoimmune Diseases, 28100 Novara, Italy

**Keywords:** Gas6, TAM receptors, Axl, MerTK, Tyro3, multiple sclerosis

## Abstract

Growth arrest specific 6 (Gas6) is a multimodular circulating protein, the biological actions of which are mediated by the interaction with three transmembrane tyrosine kinase receptors: Tyro3, Axl, and MerTK, collectively named TAM. Over the last few decades, many progresses have been done in the understanding of the biological activities of this highly pleiotropic system, which plays a role in the regulation of immune response, inflammation, coagulation, cell growth, and clearance of apoptotic bodies. Recent findings have further related Gas6 and TAM receptors to neuroinflammation in general and, specifically, to multiple sclerosis (MS). In this paper, we review the biology of the Gas6/TAM system and the current evidence supporting its potential role in the pathogenesis of MS.

## 1. The Gas6/TAM Receptors System

Growth arrest specific 6 (Gas6) is a gene firstly identified in murine fibroblasts in 1988 [1], expressed during the G0 phase and down-regulated upon induction of growth by serum. The human gene was cloned in 1993 [2] and encodes for a vitamin K-dependent protein which is expressed in different tissues, such as the gut, bone marrow, endothelial cells, and fibroblasts [2,3,4]. Structurally, Gas6 shares a high homology with protein S (ProS), another vitamin K-dependent circulating protein, which plays an anticoagulant role in vivo. Gas6 and ProS are both characterized by the presence of a C-terminal sex hormone-binding globulin (SHBG)-like structure composed by two globular laminin-G-like domains. The N-terminal region contains 11 γ-carboxyglutamic acid residuals (Gla), a loop region and four epidermal growth factor (EGF)-like domains. The post-translational carboxylation of γ-glutamyl residuals is the vitamin K-dependent process that confers a high affinity for negatively-charged membrane phospholipids, crucial for some Gas6 functions [2,5].

Gas6 and ProS are both ligands of three different tyrosine kinase receptors, collectively named TAM, an acronym for Tyro3, Axl, and MerTK; Axl is characterized by the highest affinity for Gas6 [6,7,8]. The extracellular region of the receptor consists of an immunoglobulin (Ig) domain, followed by a tandem fibronectin 3 domain; the Ig domain interacts with the SHBG-like structure of the biological ligands. The single transmembrane domain is followed by the intracellular region, which is responsible for the tyrosine kinase activity activated by receptor dimerization [9]. This is coupled to the downstream activation of different pathways, including phosphoinositide 3 kinase (PI3K)/Akt, mitogen-activated protein kinase (MAPK), extracellular signal-regulated kinases (ERK) 1/2, phospholipase C [10,11,12].

**Figure 1:**
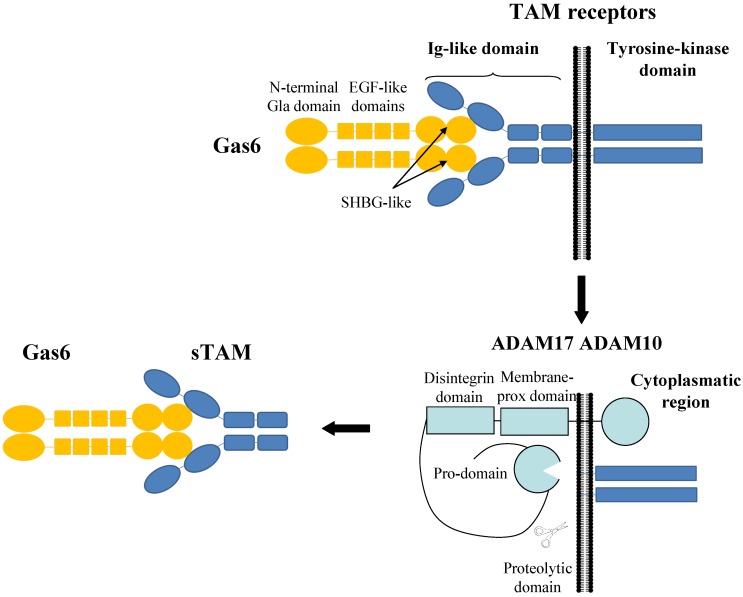
Interaction between growth arrest specific 6 (Gas6) and TAM (Tyro3, Axl and Mer) receptors. The sex hormone-binding globulin (SHBG) domain of Gas6, made of two globular laminin-G like repeats, interacts with the immunoglobulin-like domain of the TAM receptors. The extracellular portion is completed by two fibronectin 3 repeats, while the tyrosine kinase activity is played by the intracellular region of the receptor. ADAM10 and ADAM17 are two metalloproteinases responsible of TAM receptors cleavage. Their proteolytic domain cleaves TAM receptors in close proximity to the transmembrane domain, leading to the formation of soluble TAM (sTAM). sTAM receptors inhibit Gas6 activity by acting both as decoy receptors and reducing the number of ligand sites on the cell membrane (see text for further explanations and references). Ig, immunoglobulin; EGF, epidermal growth factor.

All three TAM receptors can be detected in a circulating, soluble form (respectively named sTyro3, sAxl, and sMer), which is the result of the proteolytic cleavage of the transmembrane receptor by a metalloproteinase (Figure 1) [13,14]. This cleavage results in the inactivation of the receptors; furthermore, these soluble forms act as decoy receptors for the ligands [15].

TAM receptors are differentially expressed in human tissues. Tyro3 predominates in mouse and human central nervous systems (CNS) [16,17], but it is also expressed by platelets [18], the heart [19], ovaries and testis [20], breasts [21], osteoclasts [22], and the retina [23]. Axl is widely expressed in many tissues and organs, including the brain [24], liver [25], kidney [26], heart [19], monocytes/macrophages [27], and endothelial [28] and vascular smooth muscle cells [29]. Finally, MerTK is the main mediator of Gas6 activity on immune cells [30,31], but is also expressed by the brain [24], platelets [32], gonads and prostate [33,34], lung [35], retina [23], kidney [36], and heart [19].

The Gas6/TAM system is highly pleiotropic and has many biological functions. Hence, it has been studied in many conditions. Gas6 and TAM regulate cell growth and an overactivation of the system has been associated to several neoplastic conditions and proposed as a novel therapeutic target [37,38,39,40,41,42,43,44,45,46,47,48]. Furthermore, TAM receptors are involved in haemostasis. It is well known that their ligand ProS is a master regulator of the coagulative cascade, by working as a non-enzymatic cofactor for activated protein C in the breakdown of coagulation factors (F) Va and FVIIIa [49]. Gas6 seems to play a complementary role on platelet function, which is impaired in Gas6 knockout (KO) mice, resulting in a defective thrombus formation [50].

## 2. Gas6 and TAM Receptors System, a Regulator of Innate Immunity

One of the best defined activities of the Gas6/TAM system, however, is the regulation of innate immunity. MerTK and Axl have been isolated in circulating monocytes and tissue macrophages, but not in granulocytes, T and B lymphocytes [27,51,52]. Studies on sections of spleen, lymph nodes, and thymus in mice confirmed that the mRNAs for Tyro3, Axl, and MerTK are abundant in regions populated by macrophages, but are absent in lymphocyte-rich areas [53]. Interesting lessons come from the TAM receptor KO mouse model; despite a normal phenotype of immune system at birth, within the first year of life these animals develop lympho-splenomegaly and aberrant proliferation of active T and B lymphocytes, with diffuse infiltration of tissues [53]. This constitutive activation of the immune system leads to the development of autoimmune manifestations similar to those of several human autoimmune diseases (rheumatoid arthritis, systemic lupus erythematosus, Sjögren’s syndrome, pemphigus vulgaris) and to high serum autoantibody titres [53,54]. Since lymphocytes do not express any of the TAM receptors, the splenomegaly, lymphadenopathy, and lymphocyte hyperactivation seen in TAM KO mice need to be driven by monocytes and macrophages. In fact, these cells show both an increased expression of major histocompatibility complex (MHC) class II and B7 co-receptors and an enhanced production of pro-inflammatory cytokines, including tumor necrosis factor α (TNF-α) and interleukin (IL) 12 [53].

In vitro experiments have shown that the TAM system is up-regulated when antigen-presenting cells (APCs) become activated. Toll-like receptor (TLR) activation induces the expression of Axl receptors through type I interferon (IFN) signalling, leading to suppressor of cytokine signalling proteins (SOCS) 1 and SOCS3 up-regulation, which have a critical role in switching off the inflammatory response in activated dendritic cells [55,56,57]. Consistent with these findings is the observation that the Gas6/TAM system exerts an anti-inflammatory role [58]. Gas6 is able to suppress IL-1, IL-6, and TNFα expression through the activation of MerTK-PI3K-Akt pathway in TLR-activated monocytes/macrophages, with the down-regulation of NFκB (nuclear factor kappa light chain enhancer of activated B cells) nuclear translocation [31]. Furthermore, the TAM system is involved in the regulation of type 2 immunity. In a house dust mite (HDM) murine model of allergic airway inflammation, HDM-sensitized wild-type (WT) mice developed classical signs of allergic asthma. Interestingly, HDM-sensitized *Tyro3^−/−^* mice displayed a larger increase in leukocytes and eosinophils in bronchoalveolar lavage fluid and lung, an increased infiltration of total and effector memory CD4^+^ T cells in the mediastinal lymph nodes, a higher percentages of CD4^+^ T cells producing type-2 cytokines and higher serum immunoglobulin E (IgE). This exacerbated type-2 response correlated with the lung histopathological score [59].

A second key feature of the Gas6/TAM system is the regulation of innate immunity through direct involvement in phagocytosis/efferocytosis. Again, this evidence comes from the TAM receptor single and triple mutant mice. *MerTK^−/−^* mice display a delayed clearance of apoptotic thymocytes after dexametasone stimulus, and the same occurs with the *Axl^−/−^* and *Tyro3^−/−^* single and double mutants [60,61,62]. Gas6 recognizes phosphatidylserine (PtdSer) through its amino-terminal Gla domain [63]; this lipid, normally, is expressed on the inner face of the plasma membrane but, during apoptosis, the inactivation of flippases leads to the exposure of PtdSer on the external cell membrane of apoptotic bodies [64,65]. Consequently, Gas6 bridges this lipid with TAM receptors, driving macrophages to the recognition of apoptotic cells and to their subsequent phagocytosis [54,60]. The clearance of apoptotic bodies and the production of pro-inflammatory cytokines are two tightly linked processes; in vitro, apoptotic cells, but not necrotic cells, are able to inhibit the NFκB-mediated production of pro-inflammatory cytokines by dendritic cells. Notably, MerTK binding of apoptotic bodies is required for mediating this effect. MerTK downstream cascade leads to the activation of the PI3K/Akt pathway, which inhibits IKK (IkB kinase); as a consequence, the release of NFκB from the complex with IkB is blocked, preventing its translocation to the nucleus and the transcription of the genes of pro-inflammatory cytokines, including TNF-α [66].

It is, therefore, not surprising that a dysfunction of this system has been linked to the development of autoimmune diseases, since an impaired clearance of apoptotic bodies and an inappropriate inflammatory response are considered critical for the misdirected immune response observed in these conditions.

## 3. Gas6/TAM System Regulates Survival and Functions of Neuronal and Glial Cells

In recent years a role for Gas6/TAM receptors has been postulated in the regulation of the nervous system. Gas6 is extensively expressed in the CNS [67], suggesting that interactions between Gas6 and its receptors are likely to have physiologically relevant functions [68]. All three TAM receptors are also expressed in the CNS, as reported since 1991 by Lai and Lemke [69], with Tyro3 being the most represented. The Gas6/TAM system, Tyro3 in particular, is relevant to brain development during embryogenesis. In adult mice, Tyro3 is strongly expressed by cerebral cortex and hippocampal neurons [70]; moreover, it is expressed by the amigdala, cerebellum, olfactory bulbs, and gonadotropin-releasing hormone (GnRH) neurons [71]. On the other hand, Axl and MerTK are expressed at low and constant levels during embryogenesis and adult life in mice, mainly in cerebellar and hippocampal neurons [72]; all three TAM receptors are also expressed by glial cells [73] and by endothelial and vascular smooth muscle cells in the CNS [74,75,76].

Several experiments have disclosed a role of Gas6 in promoting the survival of different neuronal cell types. In vitro, recombinant Gas6 protects hippocampal rat neurons from apoptosis induced by the deprivation of serum [77]. Moreover, Gas6 protects cortical neurons of mice from apoptosis induced by β amyloid protein and phospholipase A2 (PLA2-IIA), inhibiting chromatin condensation and DNA fragmentation. The fact that the cell cultures of these studies contained few non-neuronal cells indicates that Gas6 has a direct neuroprotective effect, not indirectly through supporting cells [78,79]. The anti-apoptotic action of Gas6 has also been described in gonadotropin-releasing hormone (GnRH) secreting neurons from mice, through the ERK cascade and PI3K [80,81]. The Gas6/TAM functional effect on adult neurons remains to be clarified; Tyro3 has been detected in clusters at dendritic, somatic, and axonal levesl but, apparently, not in synaptic connections. In view of its distribution, a role in the regulation and integration of synaptic inputs has been hypothesized; furthermore, Tyro3 might help the axonal pathfinding, being expressed by growth cones [70]. Moreover, a role in cell adhesion and cell migration had been previously suggested for Axl [72], which was identified, together with Tyro3, as a factor involved in GnRH neuronal migration along olfactory nerves from their origin in the olfactory placode to the forebrain [80]. Double KO mice for Tyro3 and Axl are characterized by a defective GnRH neuron number and migration which are responsible for impaired sexual function in female mice [71,82].

With reference to neuroglial cells, microarray analyses revealed that transcripts of tyrosine kinase Axl and MerTK receptors are expressed at high levels in isolated oligodendrocytes in the human fetal spinal cord of the second quarter [83]. The latter study also shows that human oligodendrocyte 2′,3′-cyclic nucleotide 3′-phosphodiesterase^+^ (CNP^+^) and myelin basic protein^+^ (MBP^+^) obtained from fetal spinal cord grown in the presence of recombinant human Gas6 (rhGas6) are protected from apoptosis, and develop more primary processes and arborization compared to those not treated. The effect is mediated by the Axl receptor and—downstream—by PI3K/Akt activation, and is abolished by the soluble receptor Axl-FC [83]. In a later paper by the same group a protective activity of Gas6 on TNFα-mediated cytotoxicity on human oligodendrocytes was shown, with an increase in the survival rate from 18.7% to 64.3%. This effect was Axl-dependent, being completely abrogated in oligodendrocytes derived from Axl KO mice [84]. Additionally, Gas6 stimulates the growth of human Schwann cells, increasing both the number of cells and the incorporation of tritiated thymidine, and has synergic effects with other mitogens; indeed, human Schwann cells express both Axl and MerTK and their phosphorylation is driven by Gas6 [85]. In the mice model of sciatic nerve injury, Axl is overexpressed after the nerve is damaged, again suggesting a role in the survival and protection against apoptosis [85].

Gas6 is able to regulate the inflammatory activity of glial cells, similarly to what is reported in monocytes and macrophages [31]. In 2008, Grommes et al. [86] showed that the treatment of cultured murine microglial cells with Gas6 significantly reduces the pro-inflammatory response induced by lipopolysaccharide (LPS) stimulation (IL-1β and inducible nitric oxide synthase, iNOS, are significantly down-regulated by Gas6). The Gas6/TAM system has recently been described to be relevant in physiological functions of microglia, the tissue macrophages of the brain and spinal cord. In fact, *MerTK^−/−^* and *Axl^−/−^* double-KO mice are characterized by impairment in the clearance of apoptotic bodies, reduced motility of microglial cells, and delayed recruitment to sites of brain injury; moreover, both Gas6 and ProS serve as ligands in this process [87,88].

## 4. Evidence about the Role of the Gas6/TAM System in Multiple Sclerosis: Lessons from Animal Models and Human Studies

Multiple sclerosis (MS) is an immune-mediated disorder of the CNS. The complex interactions between adaptive and innate immunity determine an inflammatory aggression to the myelin of neuronal fibers. In this context, macrophages and microglial cells are involved in myelin degradation and in oligodendrocyte loss by producing proinflammatory cytokines [89]. Therefore, systems involved in dampening macrophages activation are promising targets for studies addressing MS pathogenesis. Furthermore, in the animal model of myelin oligodendrocyte glycoprotein (MOG)-induced experimental allergic encephalomyelitis (EAE) an increased apoptosis in lymphoid organs, as well as the injection of apoptotic bodies, could worsen the disease course from a relapsing-remitting clinical pattern to a more severe secondary progressive course. However, while the underlying mechanisms have not been fully elucidated, an increase in anti-MOG antibody is observed under these circumstances [90]. Hence, impaired apoptosis also seems of significance in MS pathogenesis as it occurs for other systemic autoimmune diseases, such as systemic lupus erithematosus [91].

As mentioned before, the Gas6/TAM system has been linked to the development of autoimmunity, in the pathogenesis of which an impaired clearance of apoptotic bodies and an inappropriate inflammatory response by macrophages and dendritic cells are considered critical [54]. Additionally, the clearance of cellular and myelin debris after inflammatory demyelination might be an initial and important early step for the recovery of damaged myelin fibers; it turned out that the Gas6/TAM system is also a relevant system for both neuron and glial cell survival, including specialized cells involved in myelination processes of the CNS [83]. Thus, a role of this system in MS pathogenesis seems possible either via control of inflammation, or through the regulation of the myelination process, or both.

**Figure 2:**
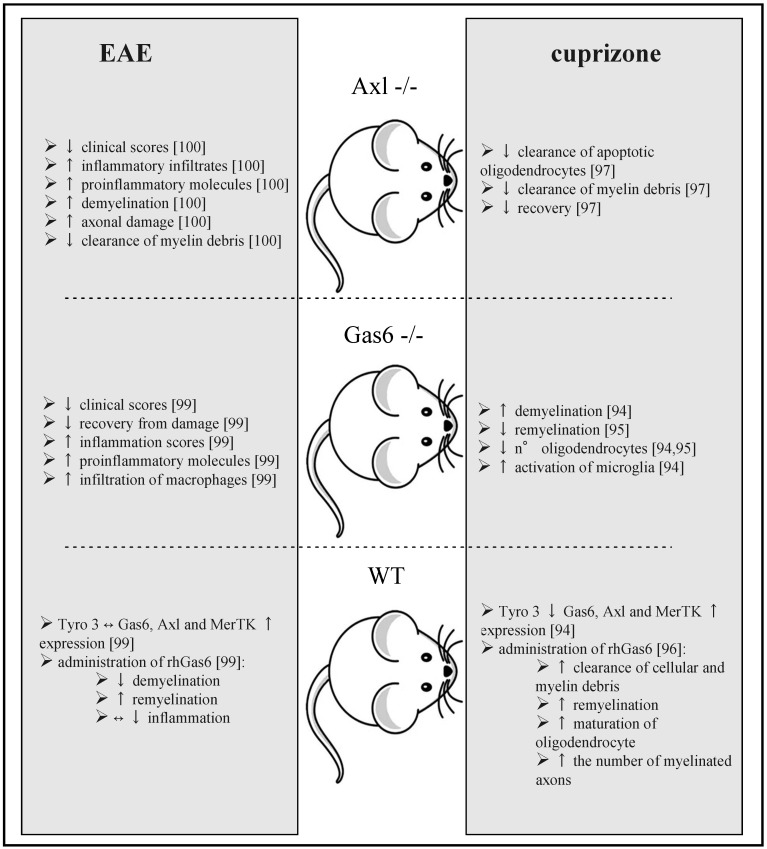
Animal models of MS involving the growth arrest specific 6 (Gas6)/TAM (Tyro3, Axl and Mer) receptors system. On the **left** are summarized data obtained in the experimental allergic encephalomyelitis (EAE) model, and on the **right** are those coming from the cuprizone challenge model, in *Axl^−/−^*, *Gas6^−/−^*, or wild-type (WT) mice, respectively. See text for further details. ↑ enhancement, ↓ reduction, ↔ no effect.

Again, the strongest evidence of Gas6/TAM system involvement in MS pathogenesis come from animal models, in particular those in which either Gas6 or TAM receptors are knocked down. Both the cuprizone demyelination and the EAE models have been used (Table 1 and Figure 2).

Cuprizone (bis-cyclohexanone-oxaldihydrazone) induces a toxic demyelination without altering the blood/brain barrier; it determines the loss of oligodendrocytes and microglial/macrophage accumulation in the damaged tissues. This model allows the study of myelin damage and repair, without the confounding factors of the intense inflammation present in mice with EAE [92,93]. In mice undergoing cuprizone challenge, a change in TAM and Gas6 expression occurs, Tyro3 is down-regulated, while Gas6, Axl, and MerTK transcription are enhanced, paralleling microglial activation. *Gas6^−/−^* mice, under the same conditions, show a more severe demyelination, a greater reduction in oligodendrocytes number, and an overactivation of microglia [94]. A further study confirmed a possible relevant role of Gas6 in myelin repair processes; in fact, *Gas6^−/−^* mice had a delayed remyelination at four weeks after cuprizone discontinuation with respect to WT, along with a reduction of oligodendrocytes. These differences, however, disappeared after 10 weeks; additionally, Gas6 significantly increased remyelination in vitro, in a dose-dependent manner [95]. Others obtained similar results. In fact, injection of rhGas6 into the CNS improved the recovery from damage after cuprizone withdrawal, with a beneficial effect on the clearance of cellular and myelin debris, enhancement of remyelination and of maturation of oligodendrocyte progenitor cells, and an increase in the number of myelinated axons [96]. The effect of Gas6 described above is mediated, at least in part, by the Axl receptor, since the clearance of damaged cells and of myelin debris, which likely impacts upon remyelination and cell survival, is impaired in *Axl^−/−^* mice. These animals have a delayed clearance of apoptotic oligodendrocytes and of myelin debris with deferred recovery from cuprizone demyelination [97]. Other in vitro and in vivo experiments link MerTK to the phagocytosis of apoptotic debris in the CNS [79,88,98].

Altogether, these data from the cuprizone mice model indicate that the Gas6/TAM receptors’ interaction is important, both during demyelination and remyelination, independently of the effects on inflammation. This system favours myelin repair after damage directly, by enhancing the clearance of cellular and myelin debris and through the support to oligodendrocyte survival and myelin restoration.

Further evidence to support Gas6/TAM involvement in the pathogenesis of MS come from the EAE model. The induction of EAE with MOG administration damages the blood-brain barrier, resulting in the infiltration of T cells and monocytes with a severe inflammation, expression of pro-inflammatory molecules, demyelination, and axonal damage. This model creates an inflammatory demyelination process similar to what happens in MS and allows studying the role of the Gas6/TAM system in neuroinflammation [92].

After EAE induction, Gas6, Axl, and MerTK RNA expression (but not Tyro3 or ProS) are significantly increased in the lumbar spinal cord; the direct intracerebral delivery of Gas6 is protective, with evidence of less demyelination and/or enhanced remyelination relative to controls. When EAE was induced in *Gas6^−/−^* mice, worse clinical scores and delayed recovery from damage were observed, and the inflammation in the spinal cord was more severe, with greater expression of pro-inflammatory molecules and a significantly increased infiltration of macrophages [99]. These data fill the gap of the previously mentioned experiments with the cuprizone model, suggesting that Gas6 is relevant in both limiting inflammatory demyelination and favouring recovery. Consistent with this hypothesis, *Axl^−/−^* mice are characterized by a more severe course of EAE than wild-type (WT) mice. Specifically, these mice develop worse spinal cord lesions with larger infiltrates, more demyelination, and more axonal damage. This is associated with larger amounts of pro-inflammatory cytokines and chemokines, such as TNFα, monocyte chemoattractant protein 1 (MCP1) and CCL5/regulated on activation, normal T cell expressed and secreted (RANTES) in the spinal cord [100]. Of note, *Axl^−/−^* mice had a strikingly impaired clearance of myelin debris by microglia/macrophages [100].

In conclusion, the data from animal models of MS altogether indicate that the Gas6/TAM system is relevant both in dampening inflammatory demyelination and in supporting myelin repair. The Axl receptor seems to be the principal effector of these actions. Thus, the Gas6/Axl interaction may be a promising target of anti-inflammatory, neuroprotective, and promyelinating treatments.

To date, very few studies replicated the above evidences in patients with MS. In 2009 Weinger et al., [101] in an autopsy study on MS patients, reported an up-regulation of sAxl and sMer in homogenates derived from chronic silent and chronic active lesions, respectively. Conversely, the full-length form of these receptors was not upregulated; intra-lesion glial cells are responsible of this expression. In normal tissue homogenates, Gas6 positively correlates with sAxl and sMer; on the contrary, in chronic active and silent lesions, Gas6 correlates inversely with sAxl and sMer. However, it is not known whether the low Gas6 concentration is due to the decoy action of sAxl and sMer leading to ligand removal or, alternatively, Gas6 secretion is reduced and, therefore, receptor cleavage enhances in the attempt to eliminate the excess of membrane-bound receptors and to restore the homeostatic ligand-to-receptor ratio. Axl and MerTK are solubilised by two metalloproteinases also called ADAM10 (a disintegrin and metalloproteinase 10) and ADAM17 (see Figure 1); in established MS lesions, the expression of these enzymes is up-regulated [101]. The shift from a positive correlation in normal tissue to an inverse correlation in established MS lesions could impair Gas6/TAM activity, affecting the functions of the system on immune response and on cell debris clearance, and favouring, in turn, a chronic demyelination environment in spite of ongoing remyelination and repair [101].

Similar observations have been reported in other human autoimmune diseases, such as juvenile systemic lupus erythematosus, where an impairment of the physiological balance between transmembrane and soluble TAM has been described. This might justify a complex derangement of the system, leading to a deficient phagocytosis and persistent inflammatory response [102].

A further clue of the association between Gas6/TAM and MS in humans has been provided by genome-wide association studies (GWAS). Several single nucleotide polymorphisms (SNPs) within the *MerTK* gene are associated with susceptibility to MS [103]. This finding was later confirmed in a large cohort of 1140 MS cases and 1140 healthy controls using a candidate gene approach. The authors identified 12 intronic SNPs related to MS susceptibility, in strong linkage disequilibrium with each other [104]. Recently, the same group reported that one specific variant of *MerTK* gene, so called rs7422195, has discordant association to MS according to HLA (human leukocyte antigen)-DRB1*15:01 status, being protective in DR15 homozygosity and favouring the disease in the absence of DR15. The minor allele of rs7422195 is also associated to an increased gene and protein expression of MerTK in monocytes and CD4^+^ cells [105]. Whether this subset of cells was formed by T cells or contaminated by monocytes expressing CD4 is not yet known. In any case, a recent study assessed the transcriptomic modification that Th17 CD4^+^ T cells undergo, derived from mice following induction of EAE. Interestingly, MerTK is among the genes overexpressed upon EAE induction [106].

Very limited evidence comes from clinical studies; our group evaluated both cerebrospinal fluid (CSF) and plasma concentration of Gas6 of MS patients by an enzyme-linked immunosorbent assay (ELISA) validated for human use and tested in other diseases and in CSF [107,108,109]. Sixty-five consecutive patients with clinically-isolated syndrome (CIS) or MS who underwent a spinal tap to confirm MS diagnosis were evaluated in relation to 45 controls affected by a non-inflammatory neurological disease. All MS patients were sampled during a relapse. The score for each functional system (FS) and the total expanded disability status scale (EDSS) score were calculated at onset, at maximum worsening, and at the first examination after the day of maximum improvement of the relapse. Relapse duration, severity, number of FS involved, and relapse recovery were obtained. We observed that patients with MS do not have substantial alterations of plasma Gas6 concentration with respect to controls, but a CSF/plasma dissociation was observed as being CSF Gas6, significantly higher with respect to other non-inflammatory neurologic diseases. Interestingly, those patients suffering a more severe or longer relapse or with more FS had a lower CSF Gas6 concentration, no different from controls, in comparison to those showing shorter and milder relapses and with fewer FS, who had significantly higher concentrations (nearly 2×). On the other hand, CSF Gas6 concentration did not change according to the completeness of recovery. Finally, neither plasma nor CSF Gas6 were related to the relapse rate or EDSS progression in a follow up cohort [110]. These findings fit with experimental evidences, according to which Gas6 is induced during demyelination in MS murine models and probably acts with a protective role in dampening neuroinflammation and in favouring myelin repair, and are in line with the report from Weinger et al., showing that Gas6 expression was very low in chronic MS lesions [101].

## 5. Conclusions

The important functions exerted in human biology by TAM receptors and their ligand Gas6, including cell growth regulation, inflammation, and clearance of apoptotic bodies, make this relatively novel system a promising target in different pathological conditions, especially if immune-driven [58]. Specifically, Gas6 and TAM seem to play a protective role against inflammatory demyelination, likely as the result of multiple mechanisms: a neurotrophic effect [24], an anti-inflammatory effect on microglia, and [86] a trophic effect on oligodendrocytes mediated by Axl [83,84], and a pro-phagocytic action, mediated by both Axl and MerTK [61]. Fewer, but consistent, evidence comes from human studies. However, the fact that Gas6 is scarcely expressed and sAxl and sMer decoy receptors are overexpressed in chronic MS lesions [101], and that the higher the CSF Gas6 concentration is, the milder the clinical MS relapse phenotype [110], are strong suggestions that activation of the TAM system in this setting is beneficial. The mechanisms by which Gas6 might exert this putative protection from the effects of inflammatory demyelination remain highly speculative; however, dysregulation of TAM cleavage is a hypothesis worthy of further testing. Indeed, a deeper understanding of the Gas6/RAM system may contribute to elucidation of MS pathogenesis and, possibly, to give us new therapeutic tools.

## Figures and Tables

**Table 1:** Summary of the current in vivo studies supporting a role for the Gas6/TAM system in multiple sclerosis.

Author	Year	Main Findings
Hoehn et al. [97]	2008	The deletion of Axl is associated with a delayed recovery and prolonged axonal damage following cuprizone toxicity
Binder et al. [94]	2008	Gas6, Axl and MerTK are upregulated upon cuprizone-induced demyelination; Gas6 knockout (KO) mice have more severe demyelination
Weinger et al. [101]	2009	In chronic multiple sclerosis (MS) lesions sAxl and sMer are upregulated and inversely related to cerebrospinal fluid (CSF) Gas6 concentration
Tsiperson et al. [96]	2010	Gas6 stimulates remyelination following cuprizone toxicity
Ma et al. [104]	2011	SNPs in MerTK gene confer susceptibility to MS
Binder et al. [95]	2011	Gas6 KO mice show a defective remyelination, after cuprizone-induced demyelination, which can be corrected by administering exogenous Gas6
ANZgene consortium [103]	2011	Many SNPs of MerTK gene are associated to MS risk in a genome wide association study
Weinger et al. [100]	2011	Axl KO murine models of experimental allergic encephalomyelitis (EAE) are characterized by a more severe phenotype than wild type mice
Sainaghi et al. [110]	2013	Gas6 CSF concentration is higher in patients with shorter and less severe MS flares
Gruber et al. [99]	2014	Intracerebral delivery of Gas6 protects against damage in EAE
Hoppmann et al. [106]	2015	CD4^+^ T cells from EAE mice show an up-regulation of Gas6 and MerTK
Binder et al. [105]	2016	SNPs in MerTK can protect or confer risk of MS on the basis of HLA-DRB1*15:01
